# Translational Organic Neural Interface Devices at Single Neuron Resolution

**DOI:** 10.1002/advs.202202306

**Published:** 2022-07-31

**Authors:** Ahnaf Rashik Hassan, Zifang Zhao, Jose J. Ferrero, Claudia Cea, Patricia Jastrzebska‐Perfect, John Myers, Priscella Asman, Nuri Firat Ince, Guy McKhann, Ashwin Viswanathan, Sameer A. Sheth, Dion Khodagholy, Jennifer N. Gelinas

**Affiliations:** ^1^ Institute for Genomic Medicine Columbia University Irving Medical Center New York NY 10032 USA; ^2^ Department of Biomedical Engineering Columbia University New York NY 10027 USA; ^3^ Department of Electrical Engineering Columbia University New York NY 10027 USA; ^4^ Department of Neurosurgery Baylor College of Medicine Houston TX 77030 USA; ^5^ Department of Biomedical Engineering University of Houston Houston TX 77004 USA; ^6^ Department of Neurosurgery Columbia University Irving Medical Center and New York Presbyterian Hospital New York NY 10032 USA; ^7^ Department of Neurology Columbia University Irving Medical Center and New York Presbyterian Hospital New York NY 10032 USA

**Keywords:** conducting polymers, human neurophysiology, neural interface devices, organic electronics, translational devices, bioelectronics

## Abstract

Recording from the human brain at the spatiotemporal resolution of action potentials provides critical insight into mechanisms of higher cognitive functions and neuropsychiatric disease that is challenging to derive from animal models. Here, organic materials and conformable electronics are employed to create an integrated neural interface device compatible with minimally invasive neurosurgical procedures and geared toward chronic implantation on the surface of the human brain. Data generated with these devices enable identification and characterization of individual, spatially distribute human cortical neurons in the absence of any tissue penetration (*n* = 229 single units). Putative single‐units are effectively clustered, and found to possess features characteristic of pyramidal cells and interneurons, as well as identifiable microcircuit interactions. Human neurons exhibit consistent phase modulation by oscillatory activity and a variety of population coupling responses. The parameters are furthermore established to optimize the yield and quality of single‐unit activity from the cortical surface, enhancing the ability to investigate human neural network mechanisms without breaching the tissue interface and increasing the information that can be safely derived from neurophysiological monitoring.

## Graphical abstract

Organic neural interface devices acquire stable neural spikes and are compatible with human neurosurgical procedures. Action potentials are detected from the surface of the human brain and clustered into putative pyramidal cells and interneurons. Design parameters to optimize the yield of single units are developed to investigate human cortical microcircuits without breaching the tissue interface.

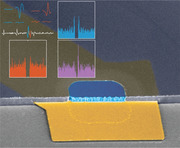

## 1 Introduction

Electrocorticography (ECoG) is a powerful tool for probing neural correlates of brain functions, identifying pathologic cortical tissue, and enabling responsive interventions in human subjects.^[^ ^1^ ^]^ These cortical surface‐based recordings of neurophysiological signals are increasingly being used for long‐term diagnostic and therapeutic purposes in patients with neurologic disorders.^[^ ^2^, ^3^, ^4^ ^]^ Conventional ECoG surveys the aggregate activity of neural populations in the form of local field potentials (LFPs), but cannot detect the action potentials of individual neurons.^[^ ^3^, ^4^, ^5^ ^]^ Despite the wealth of information available through LFP recordings of the human brain,^[^ ^6^, ^7^, ^8^, ^9^ ^]^ the ability to concurrently acquire neural spiking data has the potential to expand the utility and effectiveness of human ECoG. Recording a population of neurons with such resolution enables the identification of putative cell‐type specific responses, provides insight into neural coding during behavior, delineates recruitment into physiologic and pathologic activity patterns, and can even estimate synaptic connectivity.^[^ ^10^ ^]^ The benefits of these “single‐unit” analyses are evident through procedures that involve the implantation of penetrating electrodes into the human brain.^[^ ^11^ ^]^ For instance, the use of these approaches has elucidated neural spiking dynamics of seizures, neural encoding of high‐level percepts,^[^ ^12^ ^]^ and mechanisms of human sleep,^[^ ^13^ ^]^ in addition to forming the basis for most brain computer interfaces.^[^ ^14^, ^15^ ^]^

Microwires, jacketed sub 10 – 50 µm diameter metallic wires, are the gold standard technology to record from single neurons in the human brain.^[^ ^16^, ^17^, ^18^ ^]^ However, microwires are associated with recording instability and they have limited spatial sampling, typically of deep structures, restricting the monitoring of cortical population dynamics. Microelectrode technologies such as Utah arrays^[^ ^19^, ^20^ ^]^ or laminar electrodes^[^ ^21^ ^]^ have better spatial extent than microwire bundles, but the placement of such devices is typically restricted to areas of the cortex targeted for resection given the potential for neurologic deficit.^[^ ^22^ ^]^ Penetrating electrodes can furthermore drive a tissue immune response, thereby decreasing the longevity and function of these systems.^[^ ^23^ ^]^ The ability to record single‐unit activity from the surface of the human brain, without penetrating into tissue, could broaden clinical applicability by minimizing concern of tissue damage, recording from undisturbed superficial cortical layers, and surveying spiking dynamics over distributed cortical circuits.^[^ ^24^, ^25^ ^]^ Action potentials have been detected at the cortical surface^[^ ^26^, ^27^, ^28^ ^]^ by incorporating several design elements into recording devices: i) conducting polymers at the tissue interface, which sufficiently decrease impedance and improve signal to noise ratio; ii) electrodes sized to match the spatial scale of individual neurons (10–30 µm); iii) thin (4–6 µm), fully conformable array substrates that permit close contact with tissue.

However, challenges to the translational potential of such devices and the data they generate remain. Key device‐related requirements include optimization of signal stability and quality in a dynamic intra‐operative environment, as well as miniaturization of integrated devices to accommodate increasingly used minimally invasive neurosurgical procedures. Whether action potentials acquired with such devices can be effectively clustered and to what extent they permit examination of human cortical microcircuit features is unclear. Here, we combine technical and analytic advances to address these issues. We: i) integrated features to facilitate probe placement and long‐term stability despite the presence of circulating cerebrospinal fluid; ii) created a miniaturized, biocompatible, and serializable hard‐to‐soft electronics interface using high resolution anisotropic mixed conducting particulate composites (MCPs) to decrease physical footprint and permit signal acquisition during minimally invasive neurosurgical procedures; and iii) developed a packaging system that allows touch‐free array evaluation via impedance spectroscopy to increase procedure fidelity. To investigate the quality of data acquired by these devices, we recorded LFP and neural spiking data from human subjects undergoing implantation of electrodes for clinical deep brain stimulation. We found that spikes detected from the cortical surface by these MCP‐based NeuroGrids were amenable to clustering into single units, and we determined design parameters critical for optimizing this yield. Single units exhibited properties consistent with known cortical cell types, demonstrated microcircuit interactions at the synaptic level, and provided insight into regional population spiking dynamics. These results establish that non‐penetrating surface arrays generate high‐yield and high‐quality single‐unit activity from the human brain, opening the possibility of recording such activity from regions that are not targeted for surgical resection. They also enable advanced interrogation of neural network operations, and can be designed for compatibility with a range of clinically relevant neurosurgical procedures with potential applicability to the study of human physiology and pathology.

## 2 Results

**Figure 1:**
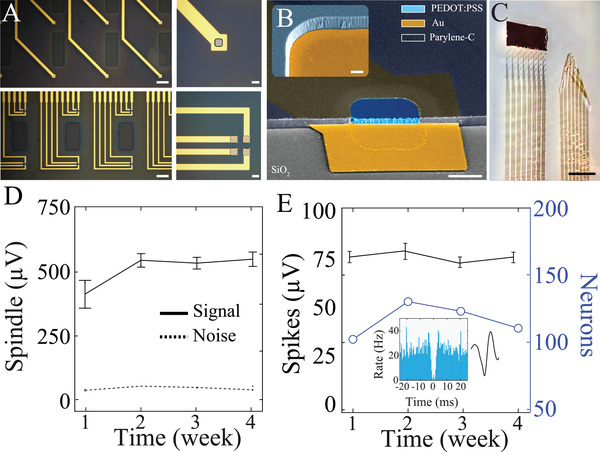
Integrated organic electronic devices for the long‐term acquisition of neural signals to the resolution of individual action potentials. A) Microscopic image of NeuroGrid configured in the single electrode or tetrode configuration, demonstrating recording electrodes interspersed with physical perforations (left; scale bars, 200 µm). Magnified microscopic image of electrodes with PEDOT:PSS coating (right; scale bars, 20 µm). B) Scanning electron cross‐sectional microscopy (SEM) image of individual PEDOT:PSS‐based electrode (scale bar, 10 µm). Inset shows a magnified image of a patterned Pa‐C side wall (scale bar, 1 µm). C) Optical image of two NeuroGrids, one with the addition of Au pad (left) and one without (right), demonstrating the utility of Au for wrinkle prevention (scale bar, 10 mm). D) Mean spindle‐band amplitude and noise floor are stable across a month of NeuroGrid recording (*n* = 3 rats). E) Mean spike amplitude (left axis) and number of putative single‐units (right axis) recorded by NeuroGrid are stable across 4 weeks (*n* = 3 rats). Inset shows mean waveform and auto‐correlogram for a sample single‐unit (bin‐size = 0.5 ms).

Surface‐based neural interfaces for long‐term recording must maintain stable, conformal contact with tissue in the presence of cerebrospinal fluid (CSF). To facilitate this contact, we harnessed material properties of electrode and substrate components within NeuroGrid arrays. Electrodes were microfabricated using PEDOT:PSS, a mixed‐conducting polymer that enables the creation of a consistently low impedance interface with neural tissue, despite contact area of 20 × 20 µm^2^ (**Figure** **1**A‐B; Figure S1, Supporting Information). These electrodes were embedded in a parylene C (Pa‐C) substrate that incorporated regularly‐spaced perforations to enable the circulation of CSF (Figure 1A). Uniform Pa‐C side‐walls and crosslinkers facilitate stability of the PEDOT:PSS – Au interface (Figure 1B, inset, Methods). To allow facile unfolding and maneuvering of the probe on the surface of the brain, a large body (8 × 4 mm^2^) of Au was incorporated at the tip of the probe to allow expansion and flattening upon contact with liquid (Figure 1C). Although not used in this manner here, this Au surface can also act as a local reference electrode for recording. We tested the effectiveness of these design elements in maintaining stable, high signal‐to‐noise ratio (SNR) neurophysiologic recordings in freely moving rats. A large craniotomy was performed, exposing the majority of the dorsal cortex of one hemisphere for NeuroGrid placement (Figure S2, Supporting Information). Implanted NeuroGrids maintained consistently low noise floor and stable amplitude of neurophysiologic signals at the level of both local field potential and putative single neuron spiking across a month of recording (Figure 1D‐E). The yield of clustered single units also remained stable across the recording epoch (Figure 1E). These results support the stability of the interface and its functional conformal contact with the brain surface in physiologic conditions, including the presence of circulating CSF.

**Figure 2:**
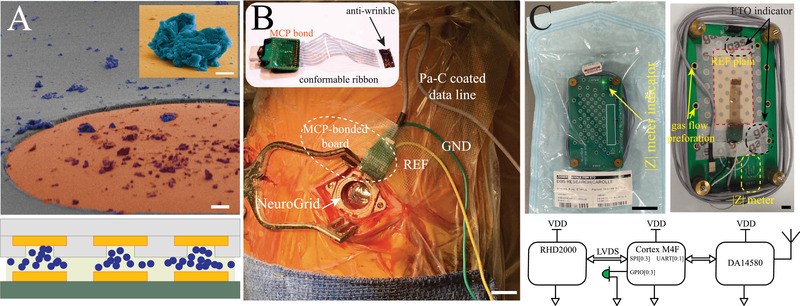
Biocompatible, sterilizable back‐end‐of‐line and functional packaging for intra‐operative recording and impedance validation. A) SEM image of MCP coating connection site (top; scale bar, 10 µm). Inset demonstrates individual MCP particles (false color; scale bar, 1 µm). Schematic of the electrode to amplifier bonding using MCP (bottom). B) Intra‐operative photograph of integrated NeuroGrid device placed within burr hole; cables for reference (REF), ground (GND), and data transmission (data line) are present (scale bar, 15 mm). Inset shows an optical image of a fully assembled device. C) A device housing unit with an embedded wireless impedance testing system inside a sterile package (top left, scale bar, 35 mm). Inside view of assembled device packaged within the housing unit equipped with impedance testing system (top right; scale bar, 15 mm). Schematic of the embedded impedance testing system with the corresponding part numbers (bottom).

A major challenge to chronic implantation and translation of such arrays is the large physical footprint at the interface between soft electronics that are in contact with the brain and electronics required for signal amplification, digitization, and transmission. Wire/stud/ball bonding have been deployed to create this interface, but these processes rely on ultrasonic‐based high thermocompression steps to establish ohmic contact between electrode pads and amplifier chip. The soft, thin substrate of conformable electronics cannot withstand these forces without mechanical reinforcement; metal layers in the contact pads must also be treated with thick (>1 µm) metals such as Ni and Au to allow the formation of Au/Au bonds across layers. Currently available anisotropic conducting films similarly require high thermocompression steps, and lack of biocompatibility (due to Ni particles and epoxy groups) limits their application to human subjects. We used mixed‐conducting particulate composites (MCP) to establish a scalable soft/hard electronics interface.^[^ ^29^ ^]^ PEDOT:PSS particles (<30 µm) blended into a biocompatible chitosan‐based adhesive^[^ ^30^, ^31^ ^]^ were deposited over contact pads using simple drop casting (**Figure** **2**A). Particle size was optimized to be larger than the height of the side walls of the printed circuit board^[^ ^32^ ^]^ (15 µm) and the Neuro Grid (2 µm) to establish elevated ohmic contacts between layers (Figure 2A; lower). This process is highly scalable and allowed direct bonding of a 250 µm pitch grid array with high reliability.

Although ECoG conventionally requires creation of a craniotomy for array placement, implantation of electrodes through small burr holes is commonly used for deep brain stimulation (DBS) and stereoelectroencephalography (SEEG) procedures. We aimed to integrate our miniaturized device with such targeted neurosurgical procedures (Figure S3, Supporting Information). Human subjects undergoing implantation of DBS electrodes for treatment of movement disorders (*n* = 7) were recruited for intra‐operative monitoring with 64‐channel NeuroGrid arrays sized to cover the cortex exposed by a typical burr hole procedure (17 mm diameter). The compact back‐end of the device allowed facile placement on the surface of the cortex intra‐operatively, and neurophysiologic recording was conducted using an epidural reference electrode (Figure 2B). As an additional safeguard, we designed a system that allowed the neurosurgeon to test the function of the complete device in the operating room immediately prior to implantation. The NeuroGrid is housed in a protective enclosure for sterilization, and we engineered an impedance testing function into this package that withstands the process of ethylene oxide gas sterilization (Figure 2C). Application of saline to the array permits impedance testing, and LED response confirms appropriate measurements from electrodes across the array. In this manner, NeuroGrids can be functionally checked in the sterile zone without jeopardizing sterility, maximizing the yield of data obtained from individual subjects.

**Figure 3:**
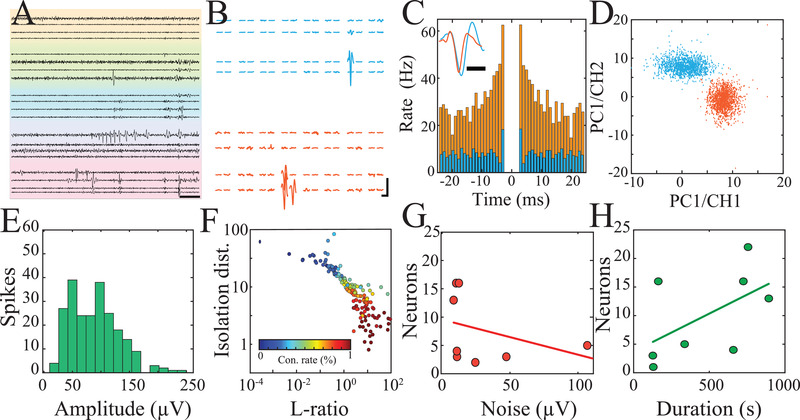
NeuroGrid enables the acquisition and clustering of putative single units from the human superficial cortex. A) The high pass filtered (250‐2500 Hz) traces were recorded by NeuroGrid from the surface of the human brain. Colors indicate signals from electrodes belonging to individual tetrodes (scale bar, 50 ms). B) Spike‐triggered average of two putative neurons with span = 1 electrode of a tetrode (blue) and span = 3 electrodes of a tetrode (orange); scale bar, 1.5 ms, 50 µV. C) Auto‐correlograms and mean spike waveforms (inset) of the two cells are shown in (B) (bin‐size = 1 ms; scale bar, 1 ms). D) Principal components (PC) of the two example cells shown in (B‐C) demonstrating separable clusters in the PC domain. E) Distribution of single‐unit waveform amplitude (*n* = 229 single‐units). F) Scatter plot of isolation distance, L‐ratio, and contamination rate (*n* = 229 single‐units). G) The yield of clustered single units is negatively correlated with the average noise floor during the intra‐operative recording session (*n* = 8 sessions across 7 subjects). H) The yield of clustered single units is positively correlated with the duration of intra‐operative recording (*n* = 8 sessions across 7 subjects).

NeuroGrids in this recording configuration were able to obtain local field potentials (LFP) and neural spiking data from the surface of human cortex in the anesthetized and awake state (**Figure** **3**A, Figure S4, Supporting Information). Detected action potentials were clustered into putative single‐units that displayed waveforms and auto‐correlograms with physiologic refractory periods (Figure 3B‐C). Neurons with overlapping action potential waveforms on the surface were separable as confirmed by principal components analysis (Figure 3D). In total, 229 putative single units were identified from 7 subjects with a cumulative recording time of approximately 4 hours. The average amplitude of all spikes detected was 88.6 ± 2.3 µV across recording sessions and the average mean waveform amplitude of single units was 93.3 ± 14.9 µV (Figure 3E). Cluster quality was evaluated using a combination of L‐ratio, isolation distance, and contamination rate (Figure 3F, Figure S5, Supporting Information). We found that the main experimental determinants of single unit yield were the duration of the session and average noise floor (Figure 3G‐H), suggesting that recording beyond the operating room environment could substantially improve the number of neurons identified from a constant number of electrodes. Therefore, we were able to cluster spiking activity into high‐quality putative single units from the surface of the intact human brain, even in regions not targeted for surgical resection.

**Figure 4:**
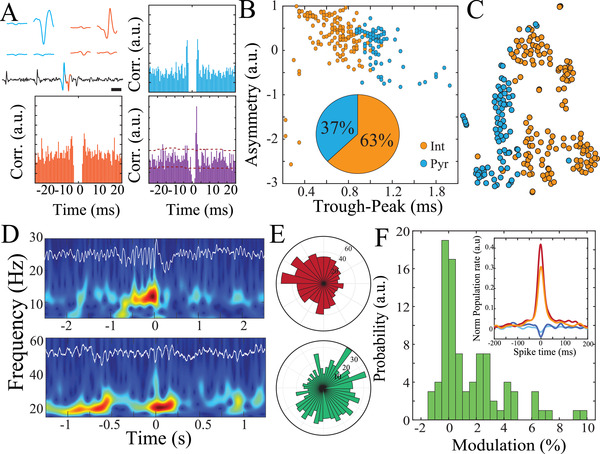
Single‐unit characterization within human cortical microcircuits. A) Spike‐triggered average waveform of a sample putative pyramidal cell (blue; upper) and interneuron (orange; upper). Action potentials of both cells appear in the high‐pass filtered time trace (scale bar, 1 ms). Autocorrelograms of these neurons (blue and orange; lower) and their cross correlogram (purple) indicates a putative monosynaptic connection (bin‐size = 0.5 ms). B) Waveform metrics roughly separate putative pyramidal cells and interneurons. Inset demonstrates the proportion of the neurons detected in all the sessions classified as putative interneurons and pyramidal cells. C) t‐Distributed Stochastic Neighbor Embedding (t‐SNE) plot (based on waveform properties, burst index, coefficient of variation) projected in two dimensions, with colors indicating classification based on waveform properties only. D) Time‐frequency spectrograms and raw traces (in white) of an example spindle‐band oscillation under anesthesia (upper) and beta oscillations during alertness (lower). E) Polar plots show phase‐locking of putative interneurons to spindle‐band oscillations (red) and pyramidal cells to beta‐band oscillations (green). F) Normalized modulation index quantifies the strength of neuron‐population coupling of human cortical neurons. Inset shows spike‐triggered population rate for four sample neurons that highlight the wide range of coupling responses for human cortical neurons.

We investigated the electrophysiologic properties of the detected single units. Waveforms and auto‐correlograms of these units had features consistent with putative pyramidal cells and interneurons (**Figure** **4**A). We were able to identify rare strongly directional cross‐correlograms at short latency (1‐3 ms) between single units suggestive of putative monosynaptic interactions and functional microcircuit membership (Figure 4A). Because putative pyramidal cells have higher trough‐to‐peak latency values and lower waveform asymmetry values than putative interneurons,^[^ ^33^, ^34^ ^]^ these features could be used to roughly classify the single units (Figure 4B). We combined waveform properties with burst index and coefficient of variation to create a high dimensional matrix that was subsequently visualized using *t*‐distributed stochastic neighbor embedding (*t*‐SNE; Figure 4C). This method revealed multiple clusters only partially separable on the basis of waveform properties, emphasizing the importance of maximizing yield of single units from human recordings to facilitate the identification of additional neural subclasses.

We next explored the relationship between single‐unit spiking and LFP patterns. In anesthetized subjects, we detected oscillations in the spindle band (Figure 4D, upper). These discrete oscillatory epochs were associated with a significant increase in neural spiking for putative pyramidal cells and interneurons on the population level, with variability in response for individual neurons. Both cell types exhibited locking of spikes to specific phases of the oscillation, with interneurons having more precise tuning (Figure 4E, Figure S6, Supporting Information). In the awake state, we identified discrete epochs of beta oscillations that strongly recruited putative pyramidal cells, but not interneurons, although both cell types displayed significant phase‐locking to these oscillations (Figure 4D, lower, 4E, Figures S7–S8, Supporting Information). Thus, interrogating the relationship between LFP and single units at the individual and/or population level is possible in the awake and anesthetized states.

To evaluate population spiking dynamics, we determined the summed activity of all the neurons in a given session and evaluated the coupling strength of individual neurons to this measure. Individual neurons had variable coupling to this population rate, ranging from strongly positive to negative correlations (Figure 4F; Figure S9, Supporting Information) and in keeping with previous observations in animal models.^[^ ^35^ ^]^ Such analyses confirm that surface‐based recording methods are capable of tracking individual and population spiking measures relevant for mapping and modulating neural networks.

**Figure 5:**
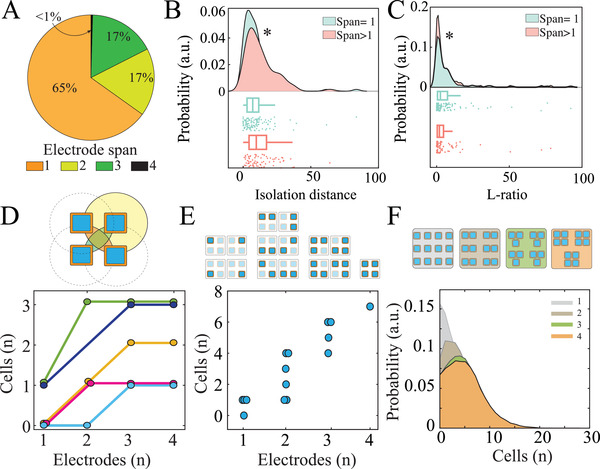
Optimization of single‐unit yield and spatial coverage with high spatiotemporal resolution ECoG. A) Proportion of single‐unit waveforms that were detected across each number of electrodes within a tetrode. B) Multi‐span cells show significantly higher isolation distance than single‐span cells (Wilcoxon rank sum test; rank sum test statistic = 4887, *p* = 0.0036). C) Multi‐span cells show significantly lower L‐ratio than single‐span cells (Wilcoxon rank sum test; rank sum test statistic = 3787, *p* = 0.04). D) Clustering quality metrics (isolation distance and L‐ratio) for multi‐electrode span single‐units undergo significant deterioration when neighboring electrodes in the tetrode were eliminated. E) Schematic demonstrating electrode span configurations for a range of 1–4 electrodes (upper). Single‐unit yield for each spatial configuration for a sample tetrode (lower). F) Schematic of spatial configurations for a sample of 12 electrodes (upper). Population histograms of single‐unit yield derived from 1000 random samplings per spatial configuration (lower). Colors correspond to spatial configurations in the schematic.

Given the physiologic variability of individual neurons in human cortex, how do we optimize yield of single units to facilitate the investigation of microcircuit mechanisms? Human ECoG must balance the trade‐off between high‐density recording for single neuron resolution and large spatial sampling.^[^ ^36^ ^]^ We hypothesized that the geometrical arrangement of electrodes would strongly affect the yield of single units. To investigate this issue, we used data derived from NeuroGrids comprised of a group of four 20 × 20 µm^2^ electrodes with 20 µm spacing between each tetrode member, and 1 mm spacing between tetrodes (**Figure** **5**A). We found that 35% of all single units were detected across multiple channels of a single tetrode; no single units spanned multiple tetrodes (Figure 5A). These multi‐channel spanning single‐units had improved cluster quality metrics compared to those in which the waveform was detected only on one channel (Figure 5B‐C). To directly test the importance of multi‐electrode detection on single‐unit yield and quality, we kept only the channel with the maximum amplitude waveform for clustering, and systematically eliminated the neighboring channels. This process resulted in an overall loss of 10% of neurons, the majority of which had initial multi‐electrode detection. In addition, multi‐electrode single‐units that were still detected post‐neighbor electrode elimination displayed poorer cluster quality metrics, including significantly decreased isolation distance and increased L‐ratio. Single units initially detected on only one electrode were relatively unaffected by electrode elimination (Figure S10, Supporting Information). Thus, we further hypothesized that an optimal number of electrodes spaced at neuron‐density could be derived to maximize single‐unit yield. For any tetrode, we determined the number of single units that would be derived from each combination of 1, 2, 3, and 4 neuron‐density spaced electrodes (Figure 5D‐E). We then randomly sampled these values 1000 times per spatial configuration, keeping the total number of electrodes constant (Figure 5F upper; 12 single electrodes, 6 groups of electrode pairs, 4 groups of triodes, and 3 groups of tetrodes). The yield of single‐units improved as number of neuron‐density spaced electrodes was increased from one to three, but then reached a plateau (Figure 5F; lower). These data suggest that groups of three neuron‐density spaced electrodes distributed across an electrode array may balance single‐unit yield with maximal spatial coverage.

## 3 Discussion

In this study, we developed integrated organic electronic devices with properties geared toward chronic implantation on the surface of the human brain, and evaluated the capacity of data generated by these devices for interrogation of cortical microcircuits at the level of isolated human single neuron activity. We demonstrated long‐term stability of the abiotic‐biotic interface, created a robust method to establish connectivity between soft and hard electronics while minimizing the device physical footprint, and streamlined the implantation process for minimally invasive neurosurgical procedures. We found that subsequently acquired data enabled i) high‐quality clustering of single‐unit activity, ii) identification of putative excitatory and inhibitory neurons as well as their circuit interactions, and iii) classification of these neurons based on their modulation by LFP patterns and population neural spiking. Furthermore, we determined specifications of array design and experimental conditions to optimize the yield of these clustered neurons. These results establish the foundation for use of such non‐penetrating neural interface devices to safely investigate human single neurons across physiologic and pathologic conditions.

Cortical interfaces capable of acquiring single‐unit data are difficult to scale for long‐term use in human subjects.^[^ ^37^, ^38^ ^]^ Biocompatible surface arrays, such as the NeuroGrid, offer the benefit of preserving an intact pial layer and thereby the potential to minimize penetration‐related damage while still providing broader coverage.^[^ ^26^, ^28^, ^39^, ^40^ ^]^ However, such devices must maintain conformal contact with the cortex in the fluid‐rich environment of the subdural space and demonstrate recording longevity to be considered for this application. We incorporated perforations into the Pa‐C substrate of the NeuroGrid to facilitate circulation and extrusion of excess CSF from the interface, and found that both LFP and neural spiking amplitudes were constant over an implantation period of at least one month. Although neural signal acquisition must be stable over much more extended time periods for brain computer interface functions, this time period is sufficient for intra‐hospital evaluation of intracranial EEG as employed during epilepsy surgery procedures.^[^ ^41^ ^]^ Another challenge to large‐scale human recordings is connectorization, as conventional approaches introduce components with substantial physical bulk and rigidity that are difficult to safely anchor and incorporate into existing neurosurgical procedures. We showed that MCP establishes the connection between soft and hard electronics on a microscopic level without introducing cross‐talk, allowing miniaturization of components and facile placement during even minimally invasive neurosurgical procedures. Therefore, MCP‐based NeuroGrids can extend solutions to some of the barriers facing large‐scale human cortical recording. Furthermore, approaches to intracranial monitoring are diversifying with the advent of stereoelectroencephalography, which employs multiple small burr holes rather than one large cranial window. Although microwires with a capacity to acquire single unit activity can be extended from the tips of these electrodes, they by nature survey predominantly deep structures rather than cortical microcircuits.^[^ ^36^ ^]^ The geometry of MCP‐based NeuroGrids is easily customizable, and we designed devices that could record to the resolution of single units from the cortical surface with only the exposure provided by a burr hole. These results support the possibility for simultaneous acquisition of neural signals from cortical depths and surface, increasing the information derived from intracranial monitoring without requiring a more extensive surgical procedure.^[^ ^42^ ^]^

We found that over one‐third of recorded neurons had action potential waveforms that were detectable across multiple 25 µm pitch electrodes on the cortical surface. In these cases, the use of closely spaced electrodes to independently sample the neuron's activity improved the yield and quality of putative single units. In contrast, action potentials with restricted spatial extent at the cortical surface were unaffected by the use of such electrode configurations. The arrangement of electrodes into groups of tetrodes also facilitated the differentiation of waveforms originating from neurons in close anatomic proximity. Improvements in action potential clustering afforded by the tetrode configuration on the surface were similar to that observed for penetrating electrodes.^[^ ^43^ ^]^ Given the cost of increasing electrode count in regard to connectorization and power efficiency of a neural interface device, our results quantify the degree to which high‐density spatial sampling of the cortical surface can benefit neural analyses based on single‐unit activity.

The ability to identify cell types from human recordings relies predominantly on the extracellular action potential waveform, as the genetic manipulations required for use of optogenetic driving or fluorescence imaging are infeasible in humans.^[^ ^44^, ^45^, ^46^, ^47^ ^]^ We were able to broadly separate single units into putative excitatory and inhibitory neurons based on the width and asymmetry of the action potential waveform, but such classifications likely underestimate the diversity of neural subtypes in the human brain.^[^ ^48^, ^49^, ^50^ ^]^ Demonstration of inhibitory/excitatory behavior as quantified by monosynaptic connectivity patterns provides more robust support for cell‐type identification. We were able to detect such monosynaptic connections from our data, but the occurrence rate was extremely low, likely related to sparse intra‐ and inter‐cortical connectivity relative to electrode sampling. Increasing the repository of human action potentials will facilitate the identification of extracellularly‐acquired features predictive of cellular classes and subclasses.

Due to the yield of single units acquired from each subject session, we were also able to compute population coupling metrics for individual neurons. We demonstrated that human cortical neurons also have a wide spectrum of coupling to population spiking. This measure predicts the responsiveness of the neuron to changes in local activity and has been demonstrated in an animal model to correspond to synaptic connectivity.^[^ ^35^ ^]^ It is of particular value to the assessment of human cortical microcircuits because it provides a proxy for anatomical features, which are essentially impossible to obtain from human subjects in vivo. Furthermore, the neural population response has been linked to response to pharmacologic agents, plasticity processes, and information processing capacity, expanding our ability to investigate human cortical dynamics despite limitations on spatial sampling and time windows for neurophysiologic recording afforded by clinical procedures.^[^ ^51^, ^52^, ^53^ ^]^

Overall, we demonstrate the material, fabrication, and analytic parameters that optimize the ability of non‐penetrating arrays to obtain high‐yield and high‐quality single unit activity from the surface of the human brain. Such approaches are critical to minimize morbidity associated with high spatiotemporal resolution neurophysiological recordings and maintain compatibility with a variety of common neurosurgical procedures. Reliable acquisition of neural spiking activity from the human cortex has the potential to shed light onto network mechanisms of higher cognitive processes and neuropsychiatric disease through multiscale investigation of the interactions between single‐unit activity and oscillatory dynamics.

## 4 Experimental Section

### Material Preparation

PEDOT:PSS solution was prepared using 20 mL Heraeus Clevios PH1000, 5 mL ethylene glycol (EG), 50 uL 4‐dodecyl benzene sulfonic acid (DBSA), and 1 wt% (3‐glycidoxypropyl) trimethoxysilane (GOPS). Micro‐90 concentrated alkaline cleaner was purchased from Special Coating Services. AZ nLOF 2020 (negative photoresist), AZ 9260 (positive photoresist), AZ 400K, and AZ 300 MIF (metal ion free) developers were acquired from MicroChemicals, Merck. Printed circuit boards were fabricated at Eurocircuits (Belgium).

### Device Fabrication

100 mm, 550 µm ± 10 µm P‐doped wafers were coated with 2 µm of parylene‐C using an SCS Labcoater 2 to form the base layer of the NeuroGrid. Metallization was patterned using a liftoff approach. AZ nLOF 2020 photoresist was spin‐coated at 3000 rpm with soft bake and post‐exposure bake at 110 °C for 90 s each. UV exposure was performed using a Suss MA6 DUV Mask Aligner and samples were developed in a bath of AZ 300 MIF. An adhesion layer of 10 nm titanium was evaporated followed by an evaporated 150 nm gold layer using a ultra‐high vacuum angstrom electron beam evaporator. Liftoff was performed using a bath of Remover PG. Subsequently, the second layer of parylene C (insulation layer), followed by an additional sacrificial layer of parylene C (for the subsequent peel‐off process) were deposited. The adhesion between the first and the second layer of parylene C was enhanced by Silane A‐174 (chemical vapor deposited) while an antiadhesion agent (5 wt% Micro‐90 diluted in DI water) reduced the adhesion between 2nd and 3rd layers. The stacked layers were patterned with a layer of AZ9260 positive photoresist and dry etched with a plasma reactive ion etching process (Oxford Plasmalab 80+; 180 W, 50 sccm (standard cubic centimeters per minute) O_2_, and 2 sccm SF_6_) to shape the electrodes and contacts. Specifically, AZ9260 was spin‐coated at 5000 rpm, baked at 115 °C for 90 s, exposed using a Suss MA6 Mask Aligner, and developed with AZ400K developer (1:4 with DI water). An extra layer of AZnLOF2020 (3000 rpm; the guard photoresist) was added between the metal layer and the later parylene layers to protect Au‐contacts from direct ion bombardment during RIE process. The contact area of the electrodes was realized by spin‐coating and baking PEDOT:PSS (600 rpm) and patterned by peeling off the sacrificial parylene layer. After patterning, the wafer is cleaned thoroughly with acetone and IPA to remove the guard photoresist and released in a water bath.

### MCP Preparation

High‐conductivity PEDOT:PSS was cast into a glass petri dish and dehydrated overnight at 120 °C. The resulting film was scraped from the dish with a stainless‐steel blade and cut into small fragments. Film fragments were suspended in IPA and crushed overnight in a bead mill using stainless steel beads ranging from 3.17 – 6.35 mm in diameter. The particle suspension was serially filtered using PET‐mesh cell strainers with pores of 1 and 10 µm in diameter. The suspension was allowed to precipitate, and excess IPA was removed. PEDOT particles were added dropwise into a 1:1 v/v chitosan (2.5 g/100 ml)‐sorbitol (40% w/v) solution under constant stirring to produce a 2.5:1 PEDOT:PSS : chitosan‐sorbitol by weight suspension.

### Backend Electronics and Device Assembly

The electronic boards are based on 100 µm thick, FR4 substrate with a 100 µm wide line (gold coated) and spacing resolution. The boards were published and smoothened using a custom‐made #2000 polisher. The exposed pads were again gold plated using an electroless gold plating solution (Transene Electronic Chemicals) at 75 °C after polishing. Boards components (Intan BGA 64‐CH amplifier chips, resistors, and capacitors were bonded to PCB using MCP and secured using medical grade silicone (MED‐1137). The complete boards were first encapsulated by 5 µm‐thick Parylene followed by a flame retardant potting epoxy curved in vacuum oven to minimize microbubbles and air cavities.

### Hard to Soft Electronic Bonding

The NeuroGrid was connected to contact pads on PCB using a high‐density MCP‐based anisotropic conductor. An adhesion layer of ACP (with low particle density) was deposited, allowed to dry for 5 min and another layer of ACP was applied. Working quickly, the grid was aligned with contact pads before ACP becomes gummy as the solvent evaporates. The impedance was tested to confirm the functionality of each channel using PBS (Sigma Aldrich, 0.01 M phosphate buffer, 0.0027 M potassium chloride, and 0.137 M sodium chloride) and an Intan acquisition board. Once attached with MCP, the outside of the parylene electrode contact region and epoxy‐coated regions are additionally coated with medical grade silicone (Advanced Silicone 40 064) to prevent rehydration of MCP during data collection and ensure biocompatibility

### Device Packaging for Sterilization

Devices including NeuroGrids, medical‐grade wires and parylene‐coated SPI cables were placed in custom housing fabricated by EuroCircuits equipped with cellulose‐based electrochemical cell, multi‐channel electrochemical impedance measurement unit, nearfield communication, and inductive coil‐based power for remote device assessment. Deviecs were secured using Ethylene Oxide indicator tape (McKesson) inside the housing. These devices were packed in two nested CrossTex DuoCheck Tyvex Pouches and sterilized with Ethylene Oxide gas at the hospital's central sterilization facility.

### Human Patient Recordings

Surgical procedures were performed at Baylor St. Luke's Medical Center (BSLMC) and Columbia University Irving Medical Center. Informed written consent from all patients was obtained, and all procedures were carried out according to protocols approved by the Institutional Review Board (IRB) at Baylor College of Medicine (BCM) and Columbia University Irving Medical Center (H‐42723). We recruited patients who were undergoing placement of deep brain stimulation electrodes to treat movement disorder (*n* = 7). Each patient underwent burr hole surgery as required for clinical diagnostics and therapeutics. The NeuroGrid was placed on the surface of the exposed cortex within the burr hole and recording was performed for a maximum duration of 26 min, at the discretion of the attending neurosurgeon and anesthesiologist. Patients were initially anesthetized, but portions of the recording occurred as the patient was being woken to participate in clinical procedures.

### Rodent Recordings

All animal experiments were approved by the Institutional Animal Care and Use Committee of Columbia University (AC‐AABI4559). The NeuroGrid implantations were carried out on the cortex of male and female Long Evans rats (*n* = 3; 8–11 weeks of age; 250–350 g) that were not used for previous experimentation. The rats were housed in pairs in a regular 12 h/12 h light/dark cycle and were separated post‐implantation. The rats were anesthetized with 2% isoflurane and the anesthesia was maintained with 0.75%–1% isoflurane during the intracranial implantation surgery. To minimize cortical oedema, methylprednisolone (30 mg kg^−1^) was administered intra‐operatively. The cortical surface was exposed by a craniotomy of the right hemisphere followed by the removal of the dura mater. The NeuroGrid arrays were placed on the cortical surface of the animals. Subsequently, the craniotomy was covered with medical silicone. After a postoperative recovery period, recordings of the neurophysiological signals were performed as the rats moved freely in their home cages.

### Data Acquisition

Neurophysiologic data was acquired using an Intan acquisition system, sampled at 20 000 Hz. Data was digitized with a local preamplifier chip and stored for off‐line analysis with 16‐bit format.

### Single‐Unit Identification

Neural spikes were detected on the basis of their amplitude using a derivative‐and‐shift peak finding and median‐based thresholding methods. Prior to any neural detection the recordings were screened (Neuroscope) and sections of the recording with prominent environmental noise were cropped from the analysis. This ensures realistic noise floor and spike template estimation. Spike clustering were done on bandpass filtered (250 – 2500 Hz) data using KiloSort.^[^ ^54^ ^]^ Manual cluster cutting to identify single units were performed and validated based on observation of spike‐triggered average waveform, auto‐correlogram, mean waveform shape, and consistency of the localization of the mean waveform with the probe geometry.

### Effect of Noise Floor and Duration on Single‐Unit Identification

The noise floor of each channel was extracted using band passed traces (*F*(*t*); 250 – 2500 Hz) using the following formula to account for cell firing variation.

(1) $$\mathrm{Noise}\;\mathrm{Floor}=\frac{\mathrm{median}\left(\mathrm{abs}\left(\mathrm{EEG}\right)\right)}{0.6745}$$

### Clustering Quality Assessment

For a cluster *K* with *n*_K_ spikes, the isolation distance was computed as the square of the Mahalanobis distance of the *n*_K_‐th closest non‐*K* spike to the center of the cluster *K*.^[^ ^55^ ^]^ If *s_i_* is the vector containing features for spike *i*, the squared Mahalanobis distance (*M* ^2^ *_i,K_*) was computed using the following formula.

(2) $$M_{i,K}^{2}={\left(s_{i}-\mu_{K}\right)}^{T}\Sigma^{-1}\left(S_{i}-\mu_{K}\right)$$

Here Σ is the covariance matrix of the action potentials in cluster *K*, and *µ_K_* is the mean feature vector of the cluster *K*. For cluster *K*, the *L*‐ratio (*L*(*K*)) was computed using the following formula.

(3) $$L\left(K\right)=\frac{1}{n_{k}}\sum_{i\notin K}\left(1-\mathrm{CDF}\times \left(M_{i,k}^{2}\right)\right)$$

Here CDF is the Chi‐squared cumulative distribution function which describes the distribution of action potentials in cluster *K*. The number of degrees of freedom of CDF was equal to the number of features used in the cluster space.^[^ ^55^ ^]^ The contamination rate was defined as the percentage of action potentials within the cluster boundary that are not from the cluster, after setting the cluster boundary at a Mahalanobis distance such that there were equal number of false negatives and false positives.^[^ ^56^ ^]^

### Cell‐Type Classification

To classify each single unit into putative pyramidal cells and interneurons, we computed the mean waveform for each of the units. Next, we extracted two waveform features: trough‐to‐peak latency and waveform asymmetry.^[^ ^33^ ^]^ Trough‐to‐peak latency is the temporal distance between the negative peak and the following positive peak of the mean spike waveform. Waveform asymmetry quantifies the relative difference of the heights of the first peak and the second peak of the mean spike waveform. A threshold was used to classify each cell as either a putative pyramidal cell or an interneuron.

### Statistics

Statistical analysis was performed using MATLAB toolboxes and custom MATLAB code. Paired t‐test was used to compare clustering quality metrics pre‐ and post‐elimination of the tetrode configuration. A modified convolution method was employed to generate the significance levels of the auto‐correlograms and cross‐correlograms.^[^ ^57^ ^]^ Rayleigh's test of nonuniformity with *κ* > 0.1 and *α* < 0.05 was used to assess if the cells had significant phase‐locking to spindle and beta oscillations.

### LFP Detection

The data between 8–20 Hz for spindle and 13–30 Hz for beta was bandpass filtered, normalized the signal, and performed envelope detection. Events were identified when the envelope was at least two std and the peak envelope was at least 3 std above the baseline. Events that were shorter than 250 ms or longer than 4 s were discarded. Visual inspection of the detected oscillations for each recording session was performed.

### Spike‐LFP Analysis

For each of the putative cells, the event‐triggered peri‐stimulus time histogram (PSTH) was generated. Data points detected as the peak power of the event were taken as PSTH reference. PSTHs with varying bin sizes (100 ms, 200 ms, and 500 ms) for consistency were validated. For spike‐LFP coupling analysis, the LFP oscillations were first filtered in the band of interest. The instantaneous LFP phase values were extracted using the Hilbert transform. Shuffling and jittering‐based statistical analysis was performed for each cell to assess the significance of spike‐LFP coupling in the spindle band. In order to compare the phase‐locking of the putative pyramidal cells and interneurons to the spindle oscillations, the preferred LFP phases of all the spikes of the respective cell types were combined.

### Population Spiking Analysis

Population rate was computed by summing all the detected single units with 1 ms resolution. Subsequently, the resulting vector was smoothed with a 50 ms Gaussian window. While computing the spike‐triggered population rate of a unit, the spikes of that unit were subtracted from the population rate. The baseline of the spike‐triggered population rate was computed by taking the average of the outer 500 ms of the 1 s window. This baseline, which reflects the baseline population rate, was subtracted to obtain the baseline‐corrected spike‐triggered population rate.

### Channel Deactivation Analysis

In order to assess the effect of electrode spacing on the performance of spike sorting, all putative cells that were detected by more than one electrode of the tetrode combination were first identified and isolated. Subsequently, all the channels of the tetrode combination except the channel where the maximum waveform was detected were deactivated. Deliberate elimination of neighboring channels was done to mimic access to only one electrode. If the cell was still detected post‐channel deactivation, cluster quality evaluation metrics before and after channel deactivation was computed. Finally, a paired *t*‐test was performed to assess the significance of the percent change of the cluster quality evaluation metrics. To further investigate if channel combination mimicking tetrodes improves the quality of unit identification, the neighboring channels of the putative cells that spanned only one channel of the tetrode configuration were deliberately eliminated. A paired *t*‐test to assess the significance of the percent change of the cluster quality evaluation metrics before and after the deactivation of neighboring channels for these cells was also performed. To derive measures of spatial yield, we used the span of each single unit's waveform across the electrodes of a tetrode and derived the number of single units generated by each combination of 1, 2, 3, and 4 neuron‐density spaced electrodes. For instance, 12 electrodes, comprising 3 tetrodes of the actual recording device, were separated into 12 individual electrodes, six groups of electrode pairs, and four groups of triodes. The single unit yield from each configuration for all tetrodes was compiled. These values were randomly sampled 1000 times per spatial configuration, keeping the total number of electrodes constant, and population histograms were generated to represent each spatial configuration.

## Conflict of Interest

The authors declare no conflict of interest.

## Author Contributions

D.K., J.N.G., conceived the project. Z.Z., C.C., P.J., and D.K. designed, developed, fabricated, and characterized the devices and materials. Z.Z., D.K., J.N.G., J.F., G.M., A.V., and S.S. performed the electrophysiology experiments. G.M., A.V., and S.S. were the attending neurosurgeons and conducted the intra‐operative recordings. J.M., P.A., and N.F. contributed to intra‐operative data acquisition. A.H. performed all aspects of neural analysis. J.N.G., D.K., Z.Z., and A.H. wrote the paper with input from all authors.

## Supporting information

Supporting Information

## Data Availability

The data that support the findings of this study are available on request from the corresponding author. The data are not publicly available due to privacy or ethical restrictions.
