# Supplementary material for: Translational Organic Neural Interface Devices at Single Neuron Resolution
Source: Adv Sci (Weinh). 2022 Jul 31;9(27):2202306. doi: 10.1002/advs.202202306 (PMC9507374; doi:10.1002/advs.202202306)
Supplement: Supplementary file 1 — Supporting Information [file ADVS-9-2202306-s001.pdf]

ADVANCED SCIENCE
Open Access

Supporting Information

for *Adv. Sci.*, DOI 10.1002/advs.202202306

Translational Organic Neural Interface Devices at Single Neuron Resolution

Ahnaf Rashik Hassan, Zifang Zhao, Jose J. Ferrero, Claudia Cea, Patricia Jastrzebska-Perfect, John Myers, Priscella Asman, Nuri Firat Ince, Guy McKhann, Ashwin Viswanathan, Sameer A. Sheth, Dion Khodagholy and Jennifer N. Gelinas**

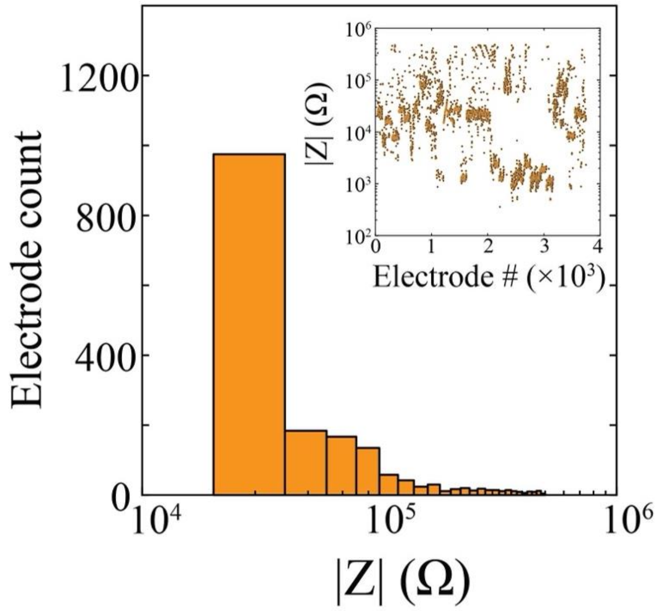

Supplementary Figure 1: High yield of NeuroGrid electrodes with functional impedance.

The majority of electrodes exhibit impedance less than 100 kΩ. Inset displays individual electrode impedance magnitudes.

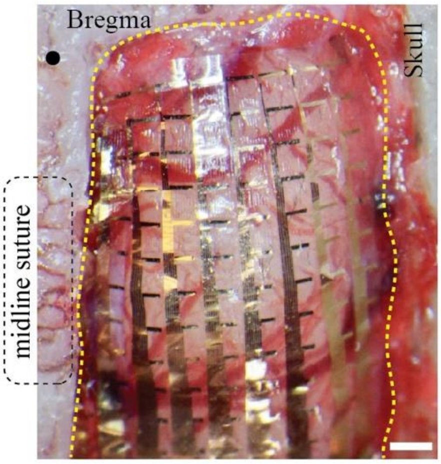

Supplementary Figure 2: Intra-operative photograph of NeuroGrid conforming to the dorsal cortical surface of the rat brain. Scale bar, 200 µm.

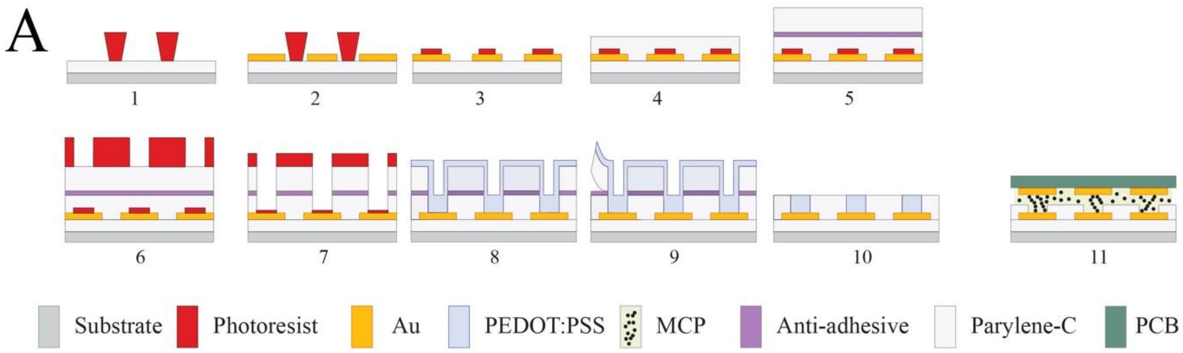

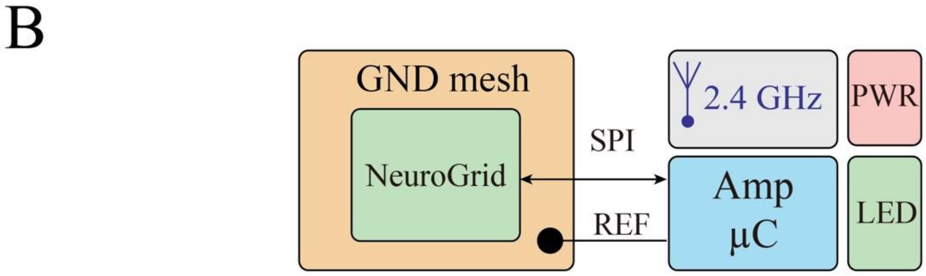

Supplementary Figure 3: Fabrication and circuit diagrams for NeuroGrid and impedance testing unit.

A) Microfabrication process steps for NeuroGrid electrodes (steps 1-10) and back-end connection with preamplifier (step 11).

B) Simplified schematic of the impedance testing unit.

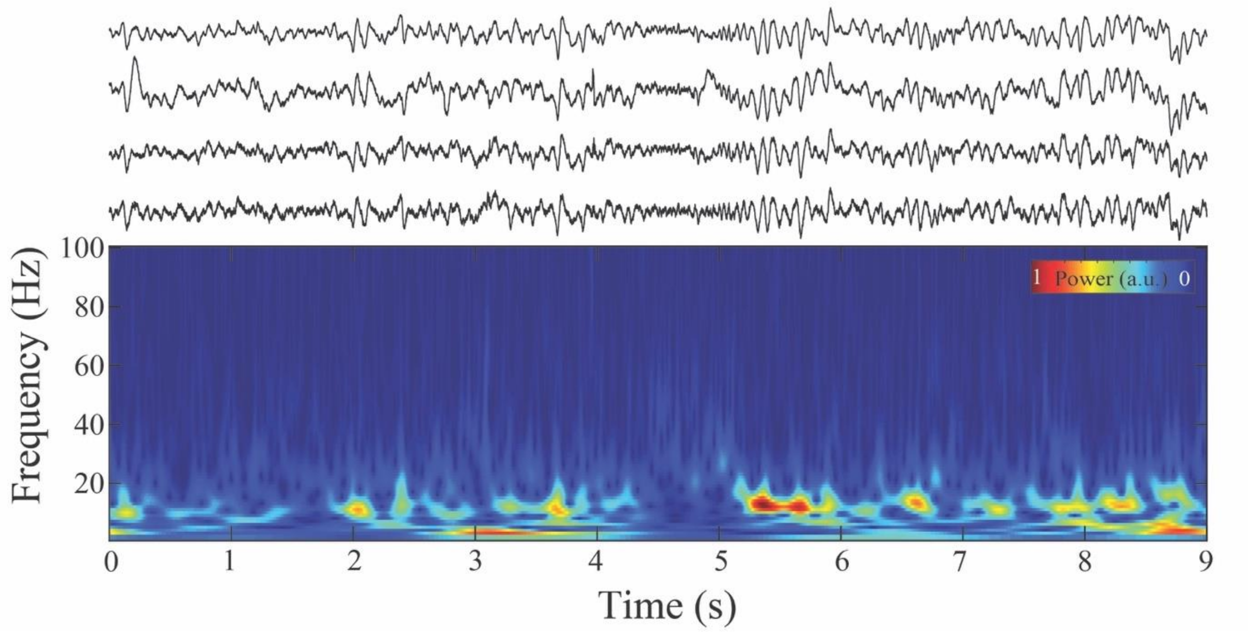

Supplementary Figure 4: Sample wideband NeuroGrid data.

A) Raw LFP traces from 4 NeuroGrid electrodes on the surface of human cortex.

B) Spectrogram of raw data in (A).

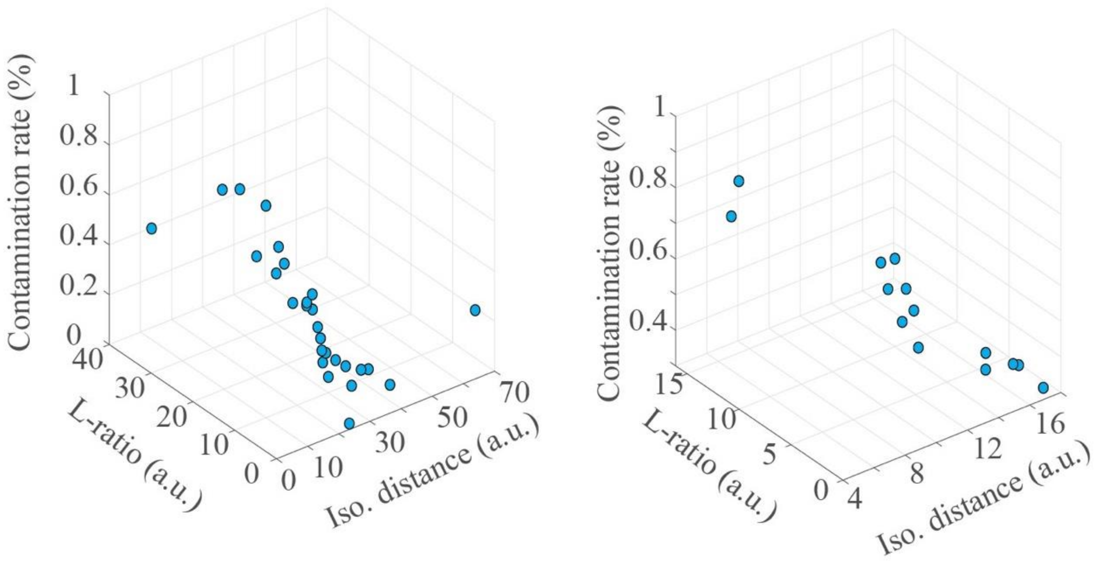

Supplementary Figure 5: Cluster quality metrics for two sample subjects.

Each dot represents one putative single-unit. Iso = isolation.

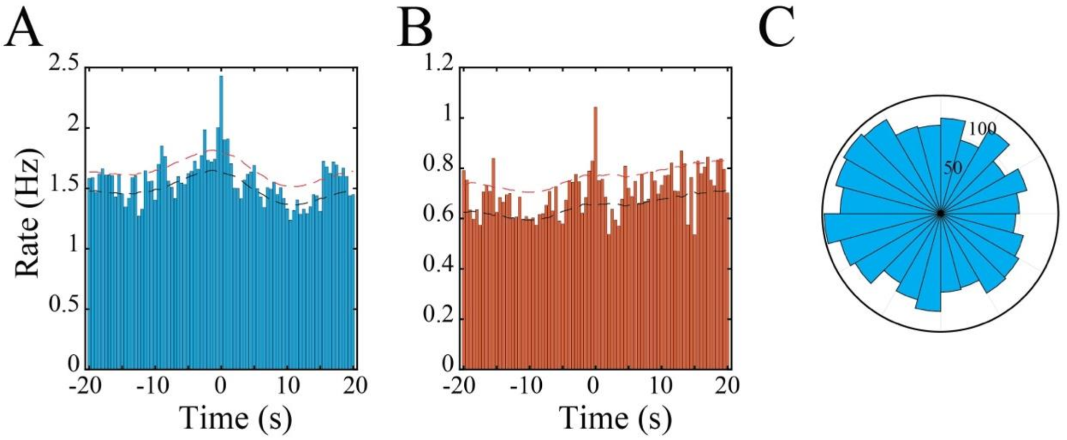

Supplementary Figure 6: Putative single-units are modulated by spindle band oscillatory patterns.

A) Peristimulus time histogram of neural spiking for pyramidal cells centered on midpoint of detected spindle-band oscillation.

B) Peristimulus time histogram of neural spiking for interneuron cells centered on midpoint of detected spindle-band oscillation.

C) Phase-locking of pyramidal cells to spindle-band oscillations under anesthesia.

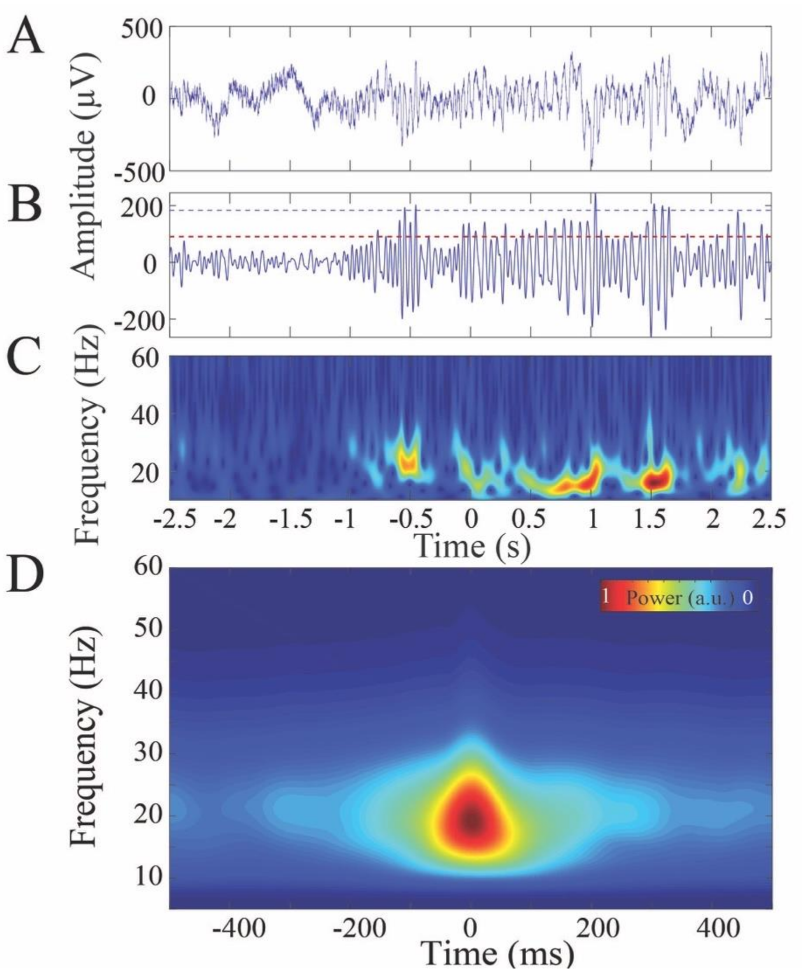

Supplementary Figure 7: Detection and characterization of beta band oscillations.

A) Raw LFP trace from a sample electrode demonstrating beta band activity.

B) Data from (A) filtered in beta band (13 - 30Hz).

C) Spectrogram of data from (A).

D) Trigger-averaged spectrogram of detected beta oscillations demonstrating peak power between 13 - 30 Hz.

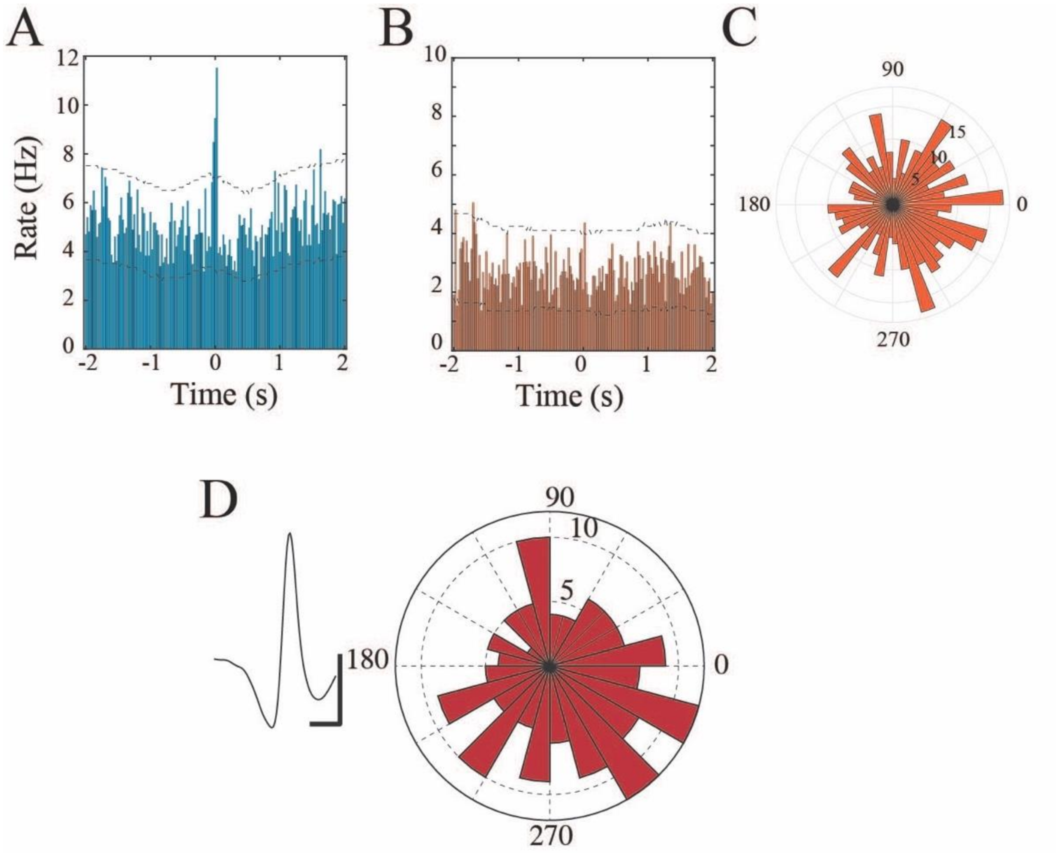

Supplementary Figure 8: Putative single-units are modulated by beta band oscillatory patterns.

A) Peristimulus time histogram of neural spiking for pyramidal cells centered on midpoint of detected beta-band oscillation.

B) Peristimulus time histogram of neural spiking for interneuron cells centered on midpoint of detected beta-band oscillation.

C) Phase-locking of interneurons to beta-band oscillations during wakefulness.

D) Sample single-unit action potential waveform (left, scale bar 0.5 ms, 50 µV) and its significant phase locking to beta band oscillations.

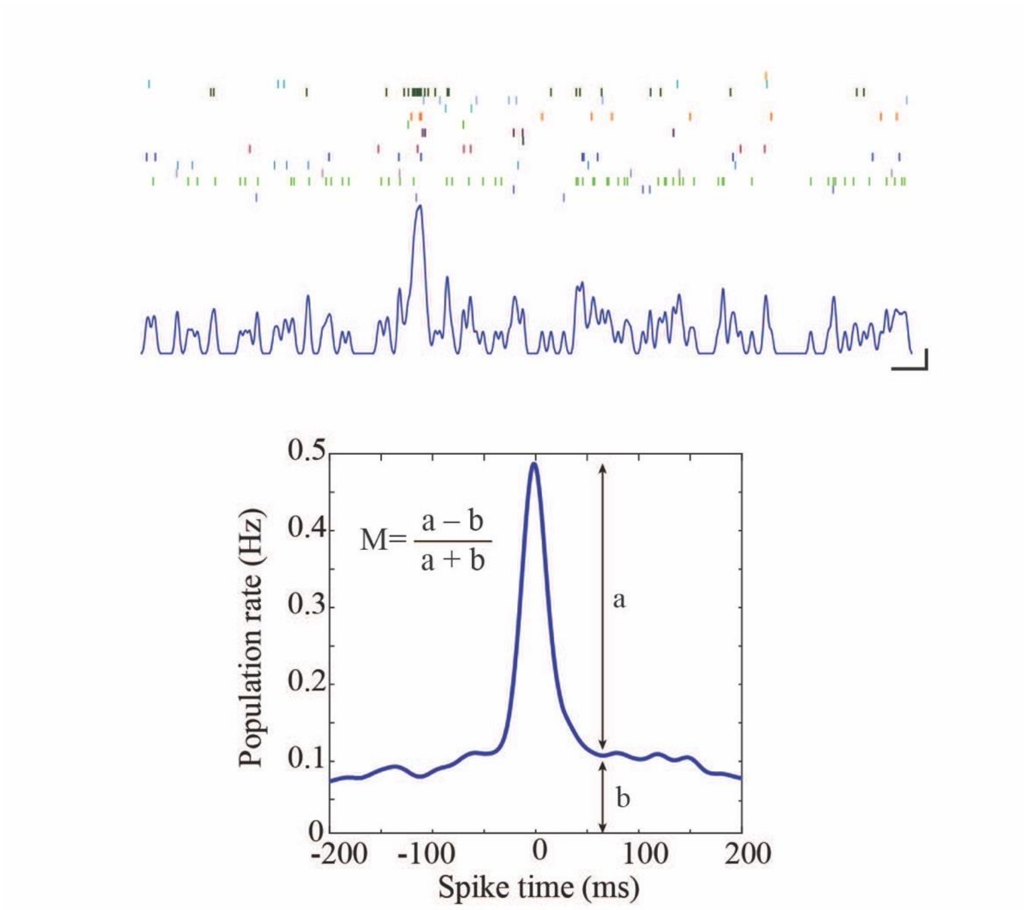

Supplementary Figure 9: Sample neural population spiking.

A) Raster plot of spiking across sample population of neurons; each color represents a different putative single-unit.

B) Summary population spiking rate for this neural population; scale bar 1 s, 1 Hz.

C) Sample population spiking quantification for a single-unit.

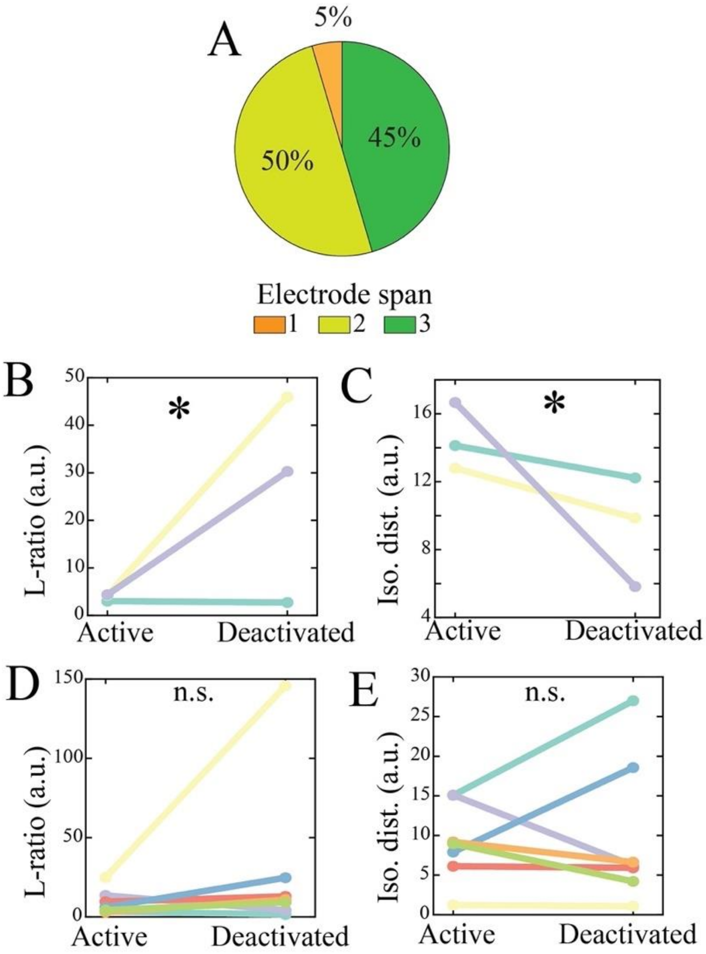

Supplementary Figure 10: Neighboring electrode elimination results in loss of single-units and poorer clustering quality.

A) Percentage of single-units spanning 1-3 electrodes that were lost when neighboring electrodes were eliminated from the clustering process.

B) Eliminating neighboring electrodes significantly increases L-ratio for multi-span single-units (t-value = 2.24, $p = 0.02$).

C) Eliminating neighboring electrodes significantly decreases isolation distance for multi-span single-units (t-value = -2.72, $p = 0.008$).

D) Eliminating neighboring electrodes does not significantly change L-ratio for single-span single-units (t-value = 1.87, $p = 0.07$).

E) Eliminating neighboring electrodes does not significantly change isolation distance for single-span single-units (t-value = 1.05, $p = 0.29$).
